# Supplementary material for: High-Magnetic-Sensitivity Magnetoelectric Coupling Origins in a Combination of Anisotropy and Exchange Striction
Source: Nanomaterials (Basel). 2022 Sep 6;12(18):3092. doi: 10.3390/nano12183092 (PMC9501851; doi:10.3390/nano12183092)
Supplement: Supplementary file 1 [file nanomaterials-12-03092-s001.zip › nanomaterials-1870841-supplementary.pdf]

nanomaterials

Supplementary Material

High-Magnetic-Sensitivity Magnetoelectric Coupling Origins in a Combination of Anisotropy and Exchange Striction

Zhuo Zeng [1,†], Xiong He [1,†], Yujie Song [1], Haoyu Niu [1], Dequan Jiang [1], Xiaoxing Zhang [1], Meng Wei [1], Youyuan Liang [1], Hao Huang [1], Zhongwen Ouyang [1], Zhenxiang Cheng [2,*] and Zhengcai Xia [1,*]

[1] Wuhan National High Magnetic Field Center & School of Physics, Huazhong University of Science and Technology, Wuhan, 430074, China

[2] Institute for Superconducting and Electronic Materials, Australia Institute for Innovation Materials, Innovation Campus, University of Wollongong, Squires Way, North Wollongong NSW 2500, Australia

* Correspondence: cheng@uow.edu.au (Z.C.); xia9020@hust.edu.cn (Z.X.)

† These authors contributed equally to this work.

Table S1. The lattice parameters of the $DyFeO_3$ single crystal.

	a	*b*	*c*	*α*	*β*	*γ*	volume
Value	5.3031	5.5983	7.6228	90.0000	90.0000	90.0000	226.3060
Sigmas	0.0001	0.0001	0.0001	0.0000	0.0000	0.0000	0.0100

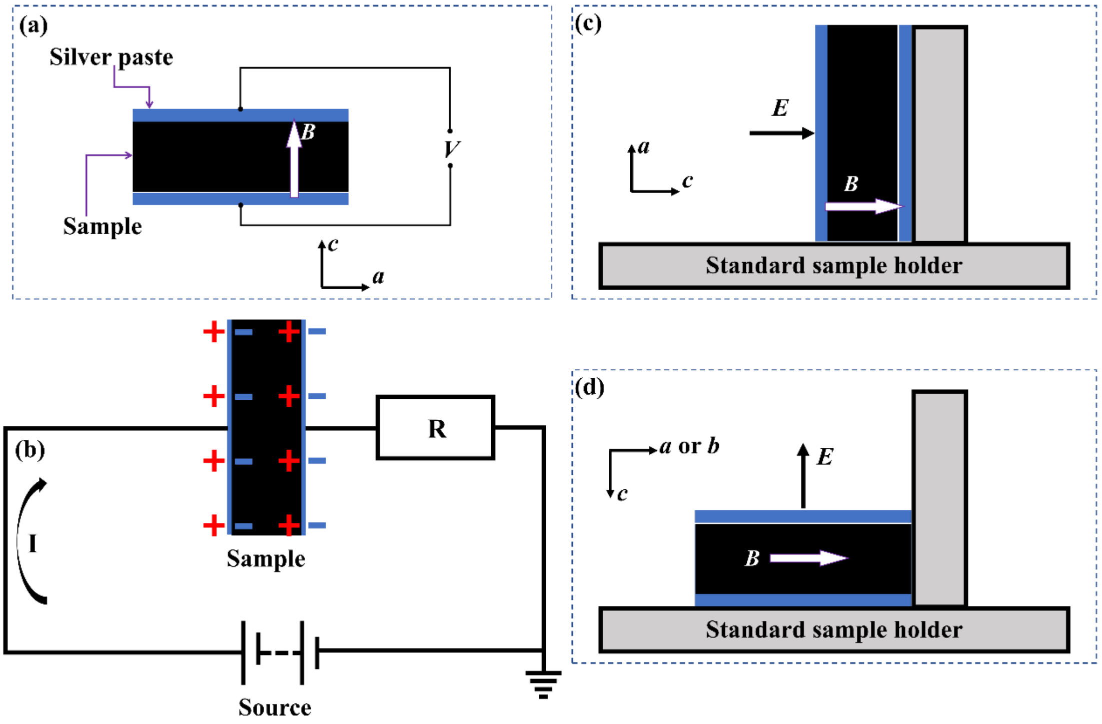

Figure S1. The measurement schematic of the electric polarization (P_c) under pulsed magnetic fields. (**a**) The schematic of the polarization measurements with the electric and magnetic fields along *c*-axis in $DyFeO_3$ sample. (**b**) Measurement circuit, the I is the current due to the changes of the field-induced polarization and R is the reference resistance used to measure the current. (**c**) The way to install sample with both of the electric field and magnetic field along *c*-axis. (**d**) The way to install sample with the electric field along *c*-axis and the magnetic field along *a*-axis (or *b*-axis).

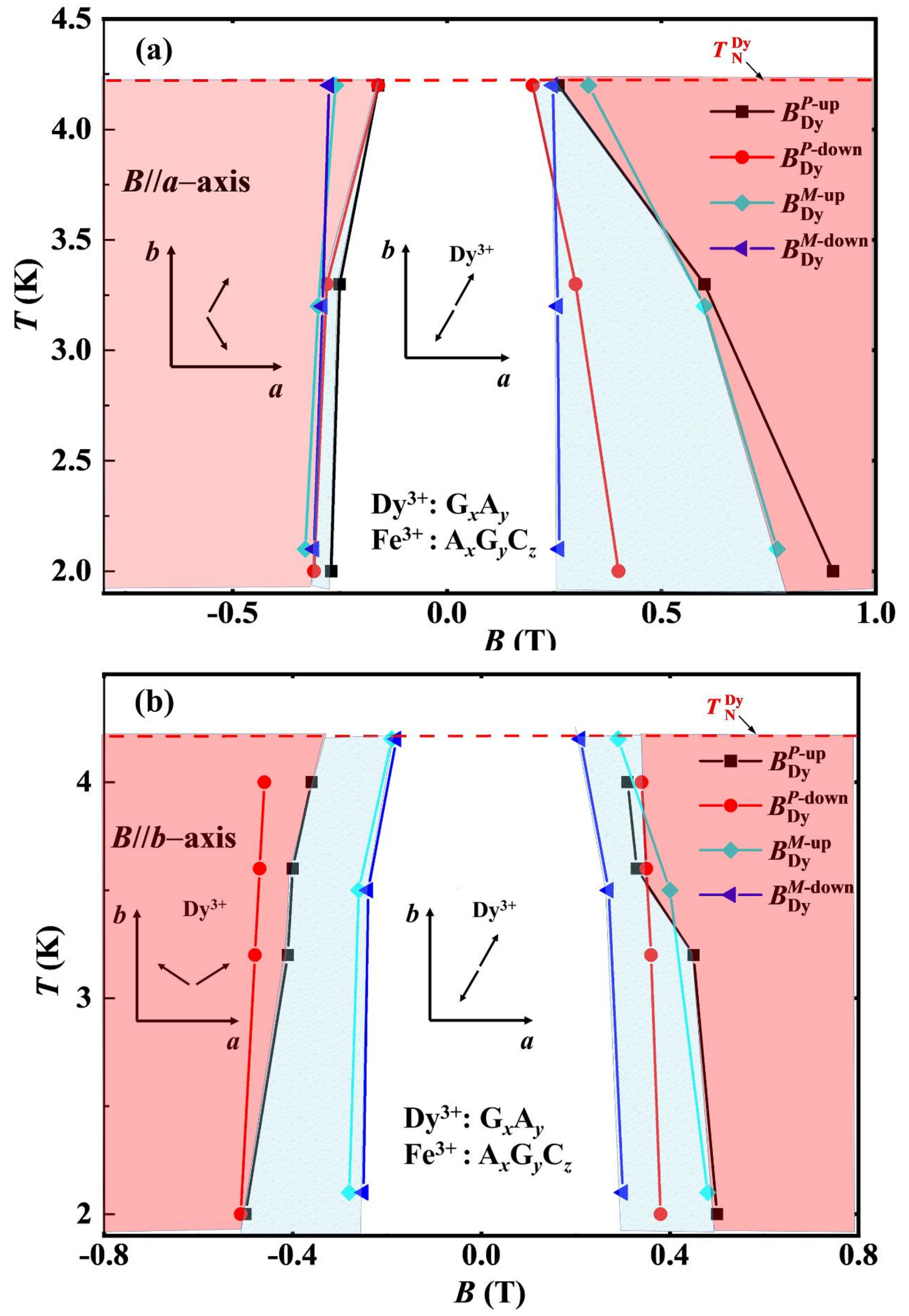
(a)
B//a–axis
Dy3+: GxAy
Fe3+ : AxGyCz
Dy3+
b
a
T N Dy
B Dy P-up
B Dy P-down
B Dy M-up
B Dy M-down
T (K)
B (T)
4.5
4.0
3.5
3.0
2.5
2.0
-0.5
0.0
0.5
1.0
(b)
B//b–axis
Dy3+: GxAy
Fe3+ : AxGyCz
Dy3+
Dy3+
b
a
T N Dy
B Dy P-up
B Dy P-down
B Dy M-up
B Dy M-down
T (K)
B (T)
4
3
2
-0.8
-0.4
0.0
0.4
0.8

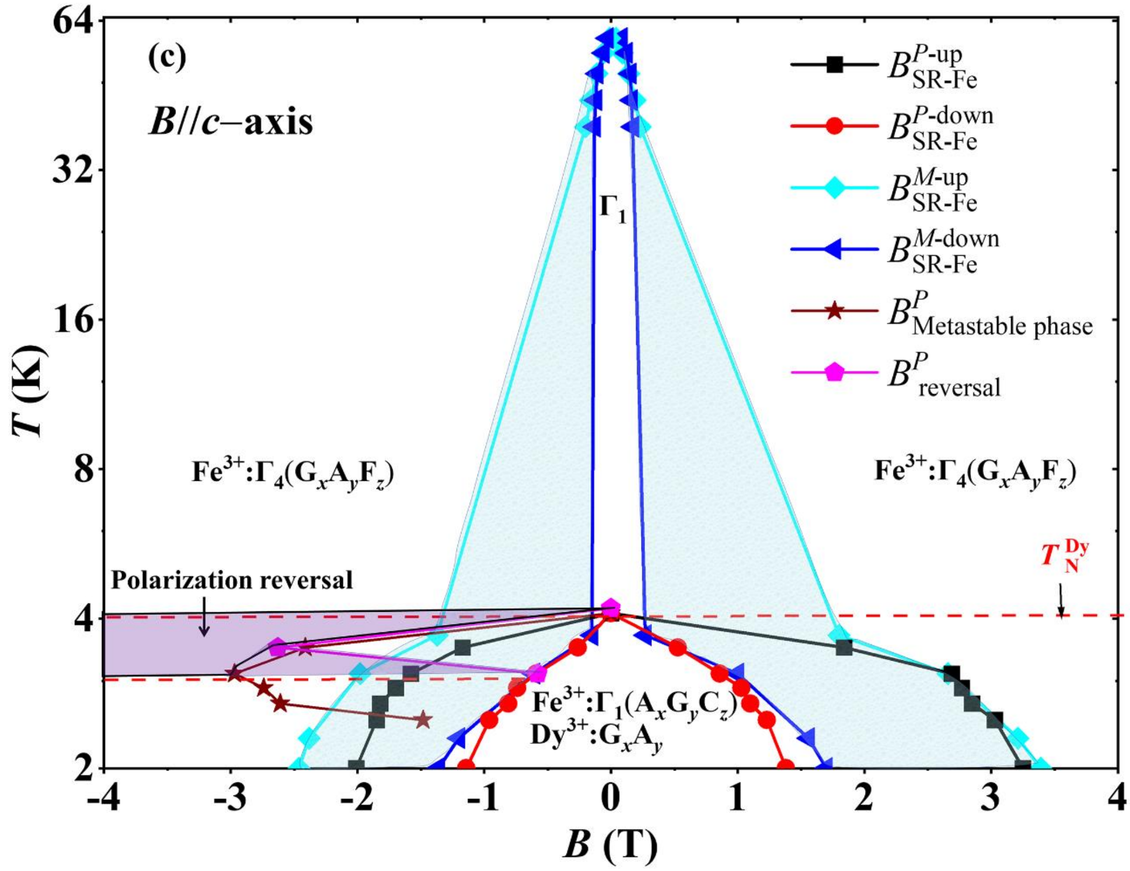

Figure S2. Magnetic phase diagrams determined with the magnetization and polarization measurements. (**a**), (**b**) and (**c**) with the pulsed magnetic field along *a*-, *b*- and *c*-axes, respectively.

With the temperature decreases to 4.2 K, Dy^{3+} spins undergo the transition from a disordered state to an ordered state with G_xA_y configuration. Applying the pulsed magnetic field along the *c*-axis can induce the spin-flop of Fe^{3+}-sublattice, and the spin configuration changes from $A_xG_yC_z$ to $G_xA_yF_z$. In this case, the moment on the Fe^{3+} layer will be parallel to the moment on the adjacent Dy^{3+} layer and antiparallel to the moment on the other adjacent Dy^{3+} layer. As a result, the Dy^{3+} layer moves towards Fe^{3+} layer due to the exchange striction and displacement polarization is generated along the *c*-axis. In Figures S2(a) and S2(b), when the magnetic field is applied along the *a*-axis, the Dy^{3+} moment rotates and leads to the $\Delta P_c \neq 0$ (along the *c*-axis) and ΔP_c has high sensitivity due to the strong magnetic anisotropy of Dy^{3+} spin. The critical fields of the transitions are basically coincident in both of the magnetization and polarization measurements. The $B_{\mathrm{Dy}}^{M\text{-up}}$ and $B_{\mathrm{Dy}}^{M\text{-down}}$ represent the critical magnetic fields in the magnetic field increasing and decreasing processes of the magnetization measurements, respectively. The $B_{\mathrm{Dy}}^{P\text{-up}}$ and $B_{\mathrm{Dy}}^{P\text{-down}}$ are associated with the critical magnetic fields in the magnetic field increasing and decreasing processes of the polarization measurements, respectively. The light red area represents the ferroelectric state, and the light green area is related to the hysteresis. When the magnetic field is applied along the *b*-axis, the magnetoelectric behaviors are similar to that of the applied magnetic field along the *a*-axis. In Figure S2(c), when the *c*-axis magnetic field is applied, the transition is related to the rotation of the Fe^{3+} moments. In addition to the previously reported transitions [1-2], there are metastable and ΔP reversal regions that may be related to the anisotropy of Dy^{3+} spin. The $B_{\mathrm{SR\text{-}Fe}}^{M\text{-up}}$ and $B_{\mathrm{SR\text{-}Fe}}^{M\text{-down}}$ represent the critical magnetic fields in the magnetic field increasing and decreasing processes of the magnetization measurements, respectively. The $B_{\mathrm{SR\text{-}Fe}}^{P\text{-up}}$ and $B_{\mathrm{SR\text{-}Fe}}^{P\text{-down}}$ are the critical magnetic fields in

the field-up and field-down processes of the polarization measurements, respectively. In the polarization measurements, the magnetic anisotropy of the Dy^{3+} spins may lead to the emergence of a metastable transition and the reversal transition. The critical magnetic fields of the metastable and reversal transitions denote as $B^{P}_{\text{Metastable phase}}$ and B^{P}_{reversal}, respectively. The blue and purple areas are associated with the hysteresis and the polarization reversal, respectively.

REFERENCES

1. Tokunaga, Y.; Iguchi, S.; Arima, T.; and Tokura, Y. Magnetic-Field-Induced Ferroelectric State in $DyFeO_3$. Phys. Rev. Lett. **101**, 097205 (2008).

2. Zhao, Z. Y.; Zhao, X.; Zhou, H. D.; Zhang, F. B.; Li, Q. J.; Fan, C.; Sun, X. F.; Li, X. G. Ground state and magnetic phase transitions of orthoferrite $DyFeO_3$. Phys. Rev. B **89**, 224405 (2014).
